# Supplementary material for: Generation and characterization of a tamoxifen-inducible, Cre driver rat for transgene expression in microglia
Source: bioRxiv. 2025 Jul 21:2025.07.17.665447. Preprint. [Version 1] doi: 10.1101/2025.07.17.665447 (PMC12330635; doi:10.1101/2025.07.17.665447)
Supplement: Supplement 1 [file NIHPP2025.07.17.665447v1-supplement-1.pdf]

bioRxiv preprint doi: https://doi.org/10.1101/2025.07.17.665447; this version posted July 21, 2025. The copyright holder for this preprint (which was not certified by peer review) is the author/funder. This article is a US Government work. It is not subject to copyright under 17 USC 105 and is also made available for use under a CC0 license.

Supplemental Figure Legends:

bioRxiv preprint doi: https://doi.org/10.1101/2025.07.17.665447; this version posted July 21, 2025. The copyright holder for this preprint (which was not certified by peer review) is the author/funder. This article is a US Government work. It is not subject to copyright under 17 USC 105 and is also made available for use under a CC0 license.

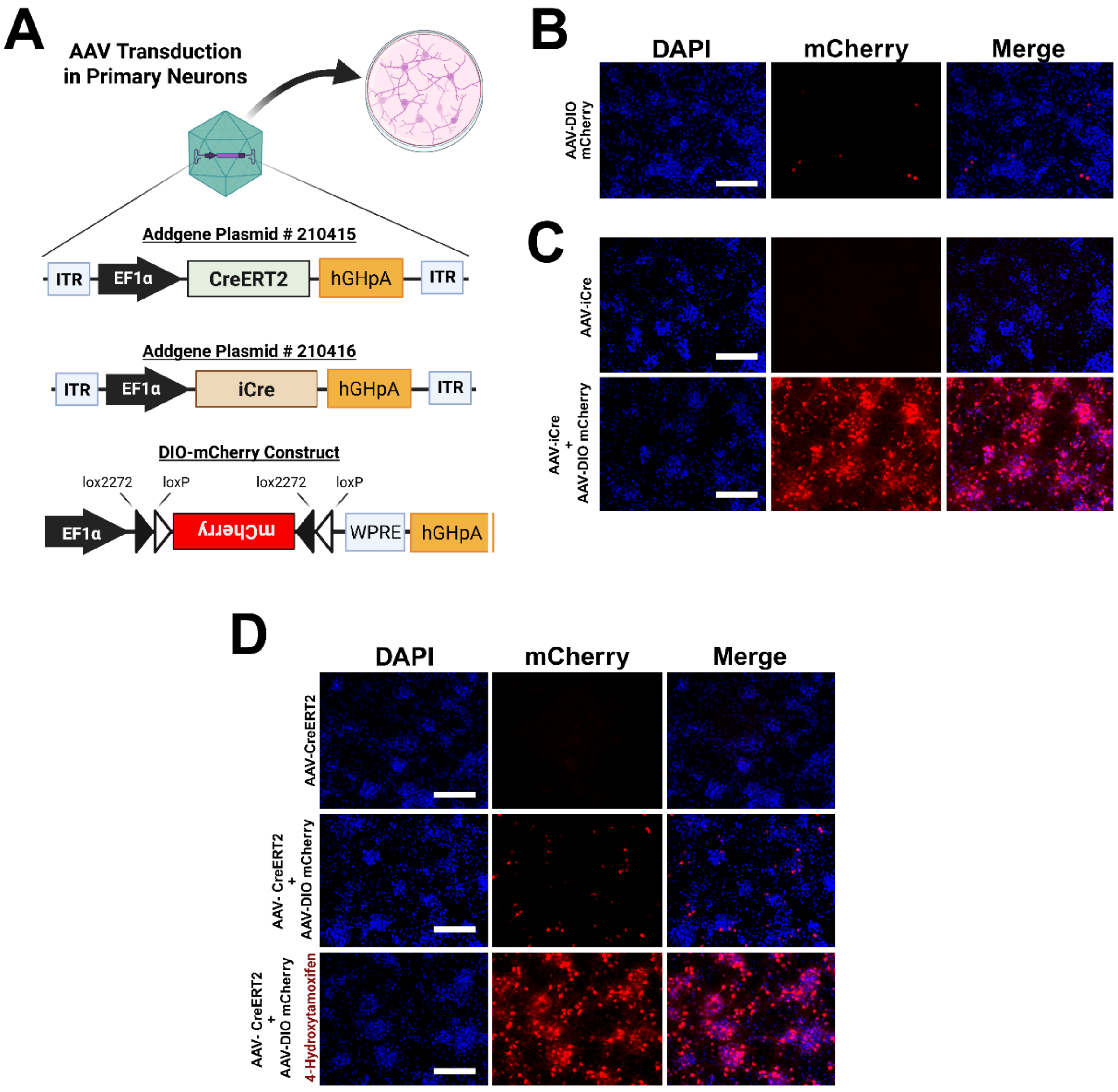

Figure S1: Verification of CreERT2 and DIO-mCherry activity prior to generating rat. **(A)** Primary cortical neurons were used to test our CreERT2 (Addgene: #210415) and DIO-mCherry constructs delivered using AAV transductions. **(B)** AAV DIO-mCherry produces minimal Cre-independent mCherry expression in PCNs. **(C)** Co-delivery of AAV-iCre (Addgene: #210416) and AAV DIO-mCherry to PCNs induces robust mCherry expression in PCNs. (D) AAV-CreERT2 and AAV DIO-mCherry co-transduction induces minimal tamoxifen-independent mCherry expression in PCNs, while addition of 4-hydroxytamoxifen (1µM) following the same AAV-transduction generates robust mCherry expression. (Scale Bar: 200 µm).

bioRxiv preprint doi: https://doi.org/10.1101/2025.07.17.665447; this version posted July 21, 2025. The copyright holder for this preprint (which was not certified by peer review) is the author/funder. This article is a US Government work. It is not subject to copyright under 17 USC 105 and is also made available for use under a CC0 license.

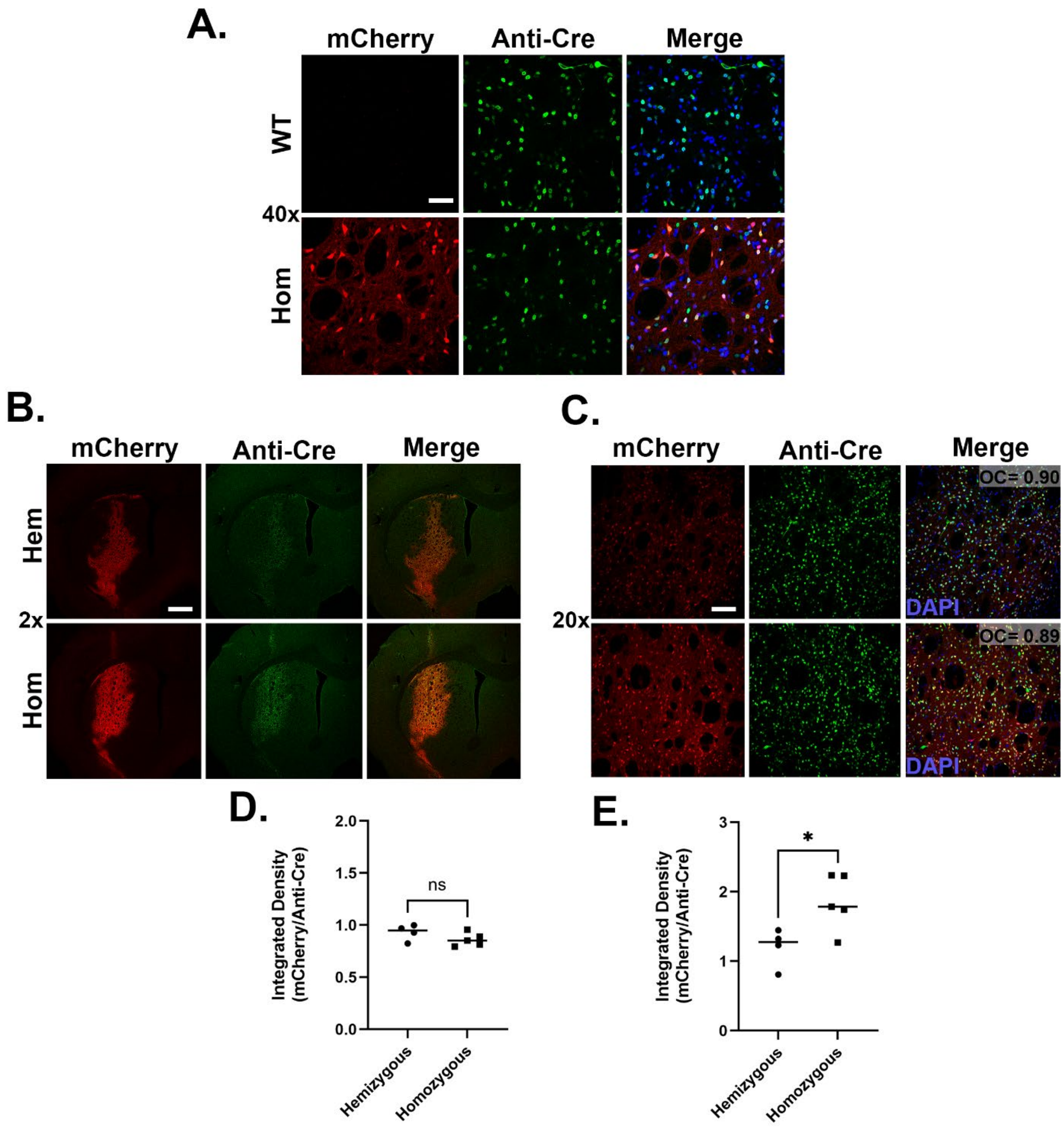

Figure S2: Hemizygous vs. Homozygous LE DIO-mCherry rat characterization. (**A**) Representative 40x magnification images from both Long Evans (LE) wildtype (WT) and DIO-mCherry (DIO-mCherry$^{+/+}$) homozygous animals two weeks after delivery of AAV-Cre into the striatum. In the presence of the AAV delivered Cre, mCherry expression is absent the WT animal and prominent in the DIO-mCherry animal. The mCherry signal is colocalized with Cre immunoreactivity (scale bar: 100 µm). (**B**) Representative low magnification images from hemizygous (Hem) and homozygous (Hom) DIO-mCherry animals 2 weeks after injections with AAV-Cre. Colocalization between Cre immunoreactivity and mCherry epifluorescence is evident in both animals (scale bar: 1 mm). (**C**) Higher magnification images from same brain sections to demonstrate colocalization of the two signals (scalebar= 200 µm)**.** Quantification of integrated density ratios of Cre immunoreactivity/DAPI fluorescent signal shows comparable expression of Cre in both Hem and Hom animals (**D**). The integrated density ratio

bioRxiv preprint doi: https://doi.org/10.1101/2025.07.17.665447; this version posted July 21, 2025. The copyright holder for this preprint (which was not certified by peer review) is the author/funder. This article is a US Government work. It is not subject to copyright under 17 USC 105 and is also made available for use under a CC0 license.

of mCherry/Cre immunoreactivity is significantly higher in the Hom animals compared to Hem animals. (**E**). Each data point represents average integrated density of two coronal sections/animal (n=4-5 per group). Unpaired t-test was used for statistical analysis (*p<0.05).

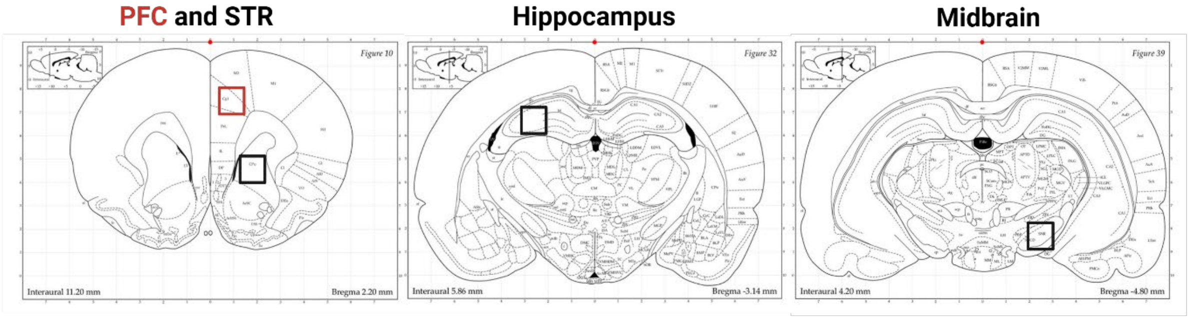

Figure S3: Brain regions where mCherry expression was analyzed for colocalization with the Iba1. Images were acquired from prefrontal cortex (PFC), striatum (STR), hippocampus (HC) and midbrain (MB). Coronal schematics were modified from Paxinos and Watson (2006).

bioRxiv preprint doi: https://doi.org/10.1101/2025.07.17.665447; this version posted July 21, 2025. The copyright holder for this preprint (which was not certified by peer review) is the author/funder. This article is a US Government work. It is not subject to copyright under 17 USC 105 and is also made available for use under a CC0 license.

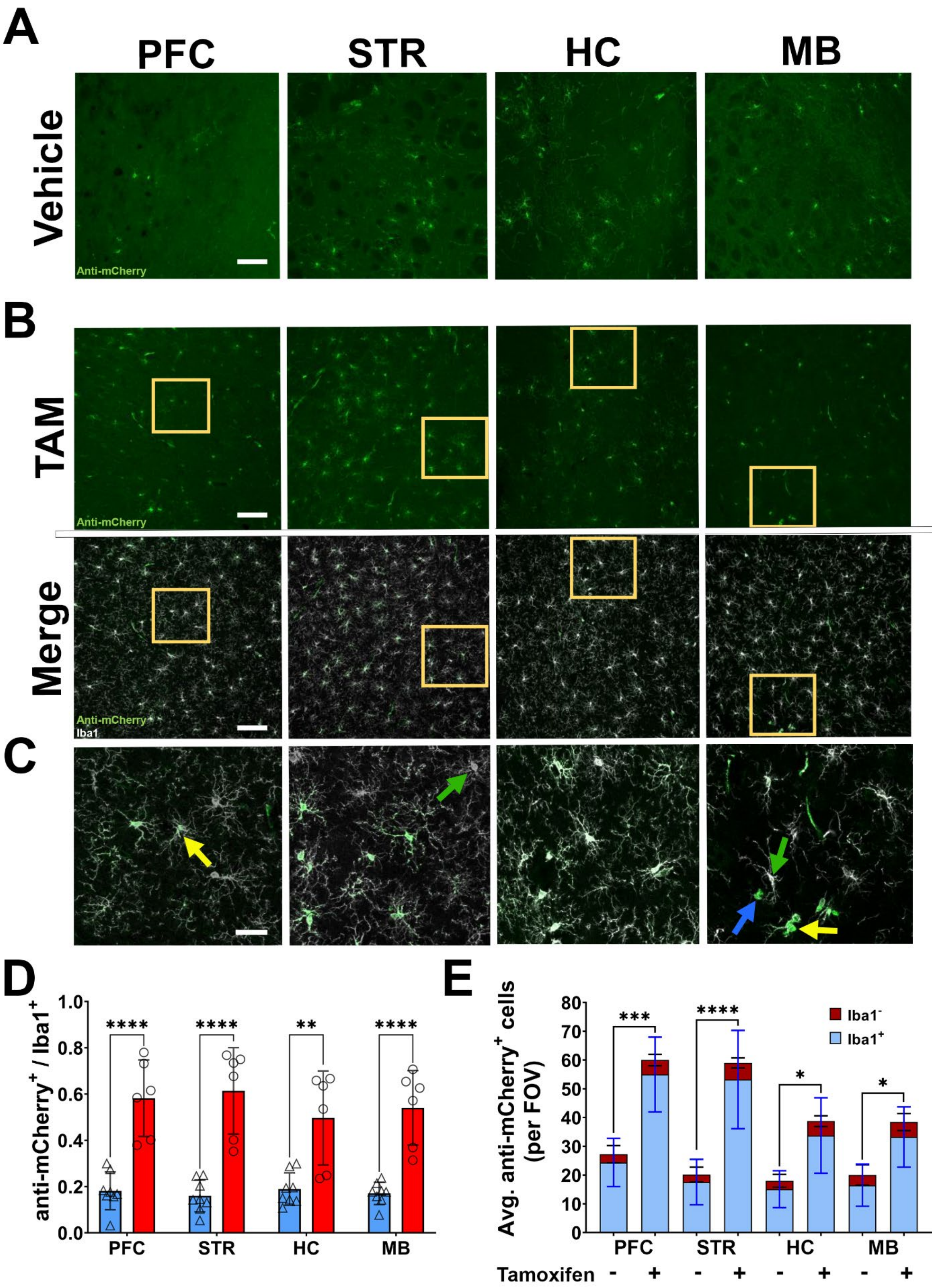

1096

bioRxiv preprint doi: https://doi.org/10.1101/2025.07.17.665447; this version posted July 21, 2025. The copyright holder for this preprint (which was not certified by peer review) is the author/funder. This article is a US Government work. It is not subject to copyright under 17 USC 105 and is also made available for use under a CC0 license.

Figure S4: Microglial Cre activity confirmation in LE Cx3cr1-CreERT2$^{+/-}$; DIO-mCherry$^{+/-}$ rat using anti-mCherry antibody. Male and female Cx3cr1-CreERT2$^{+/-}$; DIO-mCherry$^{+/-}$ rats received three i.p. injections of either tamoxifen (TAM) (male, n=3; female, n=4) or vehicle (male, n=4; female, n= 3) at P10. Brain tissue was collected 8 weeks after injections. Brains were sectioned and images taken proximal to the prefrontal cortex (PFC), striatum (STR), hippocampus (HC), and midbrain (MB) sections**.** Representative regional images show anti-mCherry (green) expression in vehicle **(A)** and TAM **(B)** injected rats (scale bar:100 µm) (top panel of **B**). Colocalization of anti-mCherry (green) and Iba1 (white) (Merge) in TAM injected rats is shown in the Merge panel of **(B)**. Regions shown at higher magnification in **(C)** (scale bar= 30 µm) are highlighted in yellow boxes from **(C)**. Yellow arrows indicate representative Iba1^{+} anti-mCherry^{+} cells; green arrows highlight Iba1^{+} anti-mCherry^{-} cells; blue arrows show Iba1^{-} anti-mCherry^{+} cells. **(D)** Ratio of anti-mCherry^{+} /Iba1^{+} cells in vehicle (n=7) and tamoxifen (n=6) treated rats (male and female animals combined). **(E)** Raw counts of mCherry^{+} cells in vehicle and tamoxifen treated animals. Each bar indicates average quantity of anti-mCherry^{+} cells that are Iba1^{+} (blue) and Iba1^{-} (red) per field of view. Two-way ANOVA with Tukey's multiple comparison with S.D. error bars (**p<0.01; ***p<0.001; ****p<.0001).

bioRxiv preprint doi: https://doi.org/10.1101/2025.07.17.665447; this version posted July 21, 2025. The copyright holder for this preprint (which was not certified by peer review) is the author/funder. This article is a US Government work. It is not subject to copyright under 17 USC 105 and is also made available for use under a CC0 license.

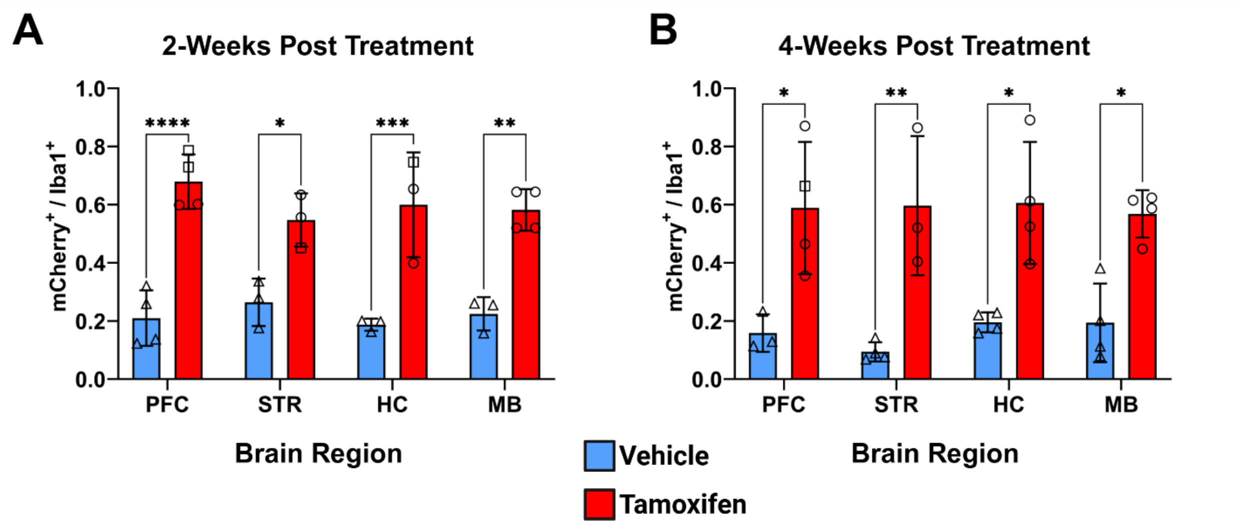

Figure S5: Two- and four-week post tamoxifen mCherry^{+}/Iba1^{+} cell count ratios in the LE Cx3cr1-CreERT2$^{+/-}$; DIO-mCherry$^{+/-}$ rat brain. Male and female double transgenic animals (Cx3cr1-CreERT2$^{+/-}$; DIO-mCherry$^{+/-}$) received three-five i.p. injections of either tamoxifen (TAM) (male, n=3; female, n=4) or vehicle (male, n=4; female, n= 3) at P10. Brain tissue was collected 2- or 4- weeks after vehicle or tamoxifen injections. Brains were sectioned and images taken proximal to the prefrontal cortex (PFC), striatum (STR), hippocampus (HC), and midbrain (MB)**.** Square symbols indicate animal was given five TAM injections. Two-way ANOVA with Tukey's multiple comparison (*p<0.05; **p<0.01; ***p<0.001; ****p<.0001) with error bars indicating S.D.

bioRxiv preprint doi: https://doi.org/10.1101/2025.07.17.665447; this version posted July 21, 2025. The copyright holder for this preprint (which was not certified by peer review) is the author/funder. This article is a US Government work. It is not subject to copyright under 17 USC 105 and is also made available for use under a CC0 license.

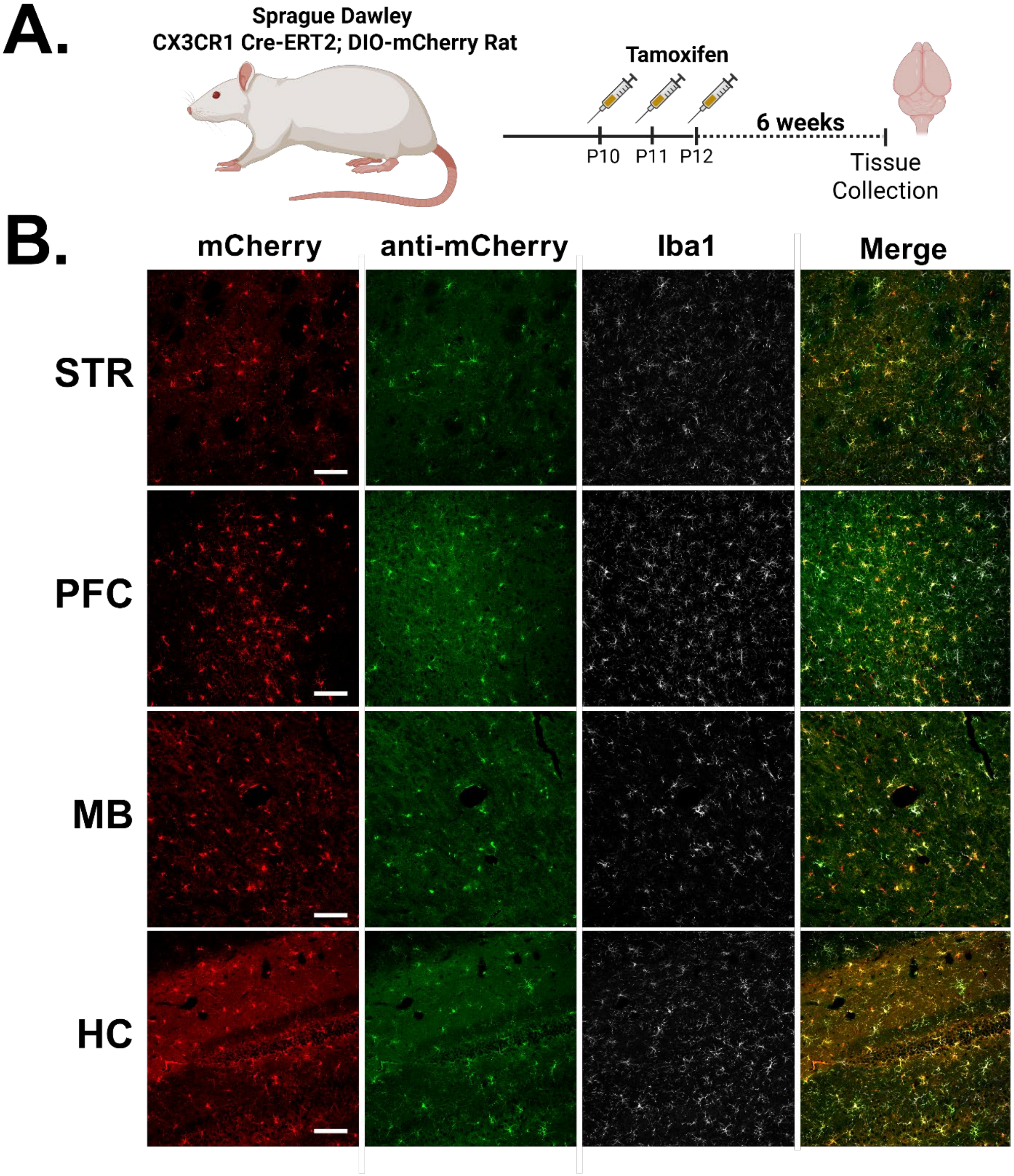

Figure S6: Specificity of microglial Cre activity in SD background Cx3cr1-CreERT2$^{+/-}$; DIO-mCherry rat$^{+/-}$. **(A)** Sprague Dawley Cx3cr1-CreERT2; DIO-mCherry rats were injected with TAM (60mg/kg) once daily for three days. Brains were collected 6 weeks post TAM, sectioned and analyzed for ectopic mCherry expression (red) and anti-Iba1 (white) and anti-mCherry (green) immunoreactivity in the prefrontal cortex (PFC), striatum (STR), hippocampus (HC), and midbrain (MB) **(B)**. Colocalization of all three channels visualized in the "Merge" column. Scale Bar =100 µM

bioRxiv preprint doi: https://doi.org/10.1101/2025.07.17.665447; this version posted July 21, 2025. The copyright holder for this preprint (which was not certified by peer review) is the author/funder. This article is a US Government work. It is not subject to copyright under 17 USC 105 and is also made available for use under a CC0 license.

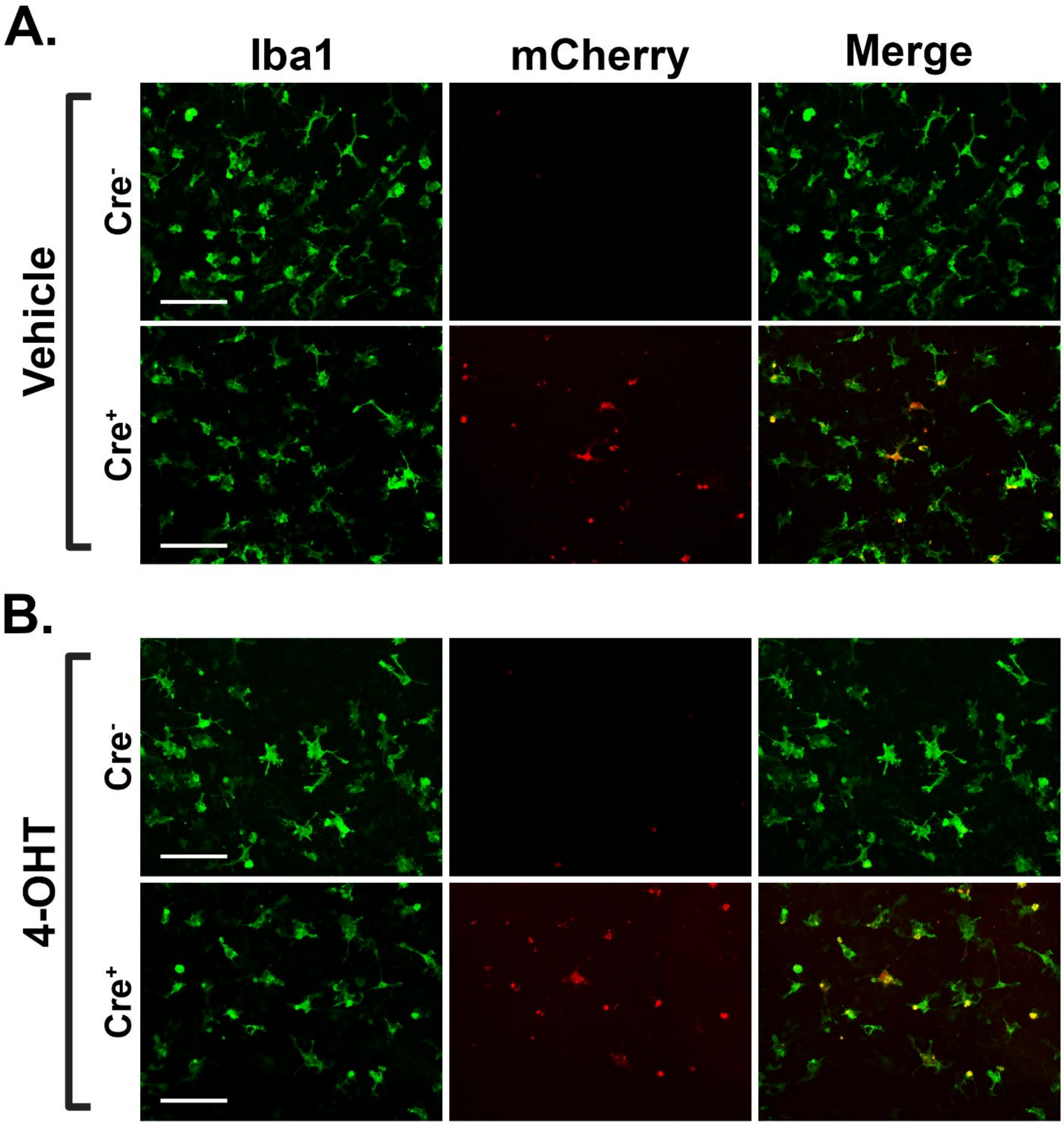

Figure S7: Primary Microglia from SD background Cx3cr1-CreERT2$^{+/-}$; DIO mCherry$^{+/-}$ animals. Primary microglia were isolated and plated from 2 days post-natal pups of Cx3cr1-CreERT2$^{+/-}$ × DIO-mCherry$^{+/-}$ crossing. Genotyping was performed from postmortem tissue collection. Cells were treated with either Vehicle (**A**) or 4-hydroxytamoxifen (4-OHT) (**B**). mCherry expression was not detected in microglia from animals without the CreERT2 (Cre^{-}) transgene in either the Vehicle or 4-OHT treatment groups. Vehicle treated microglia with the CreERT2 (Cre^{+}) show mCherry expression, indicative of basal levels of active Cre (**A**). mCherry signal is increased in Cre^{+} microglia treated with 4-OHT (**B**).

bioRxiv preprint doi: https://doi.org/10.1101/2025.07.17.665447; this version posted July 21, 2025. The copyright holder for this preprint (which was not certified by peer review) is the author/funder. This article is a US Government work. It is not subject to copyright under 17 USC 105 and is also made available for use under a CC0 license.

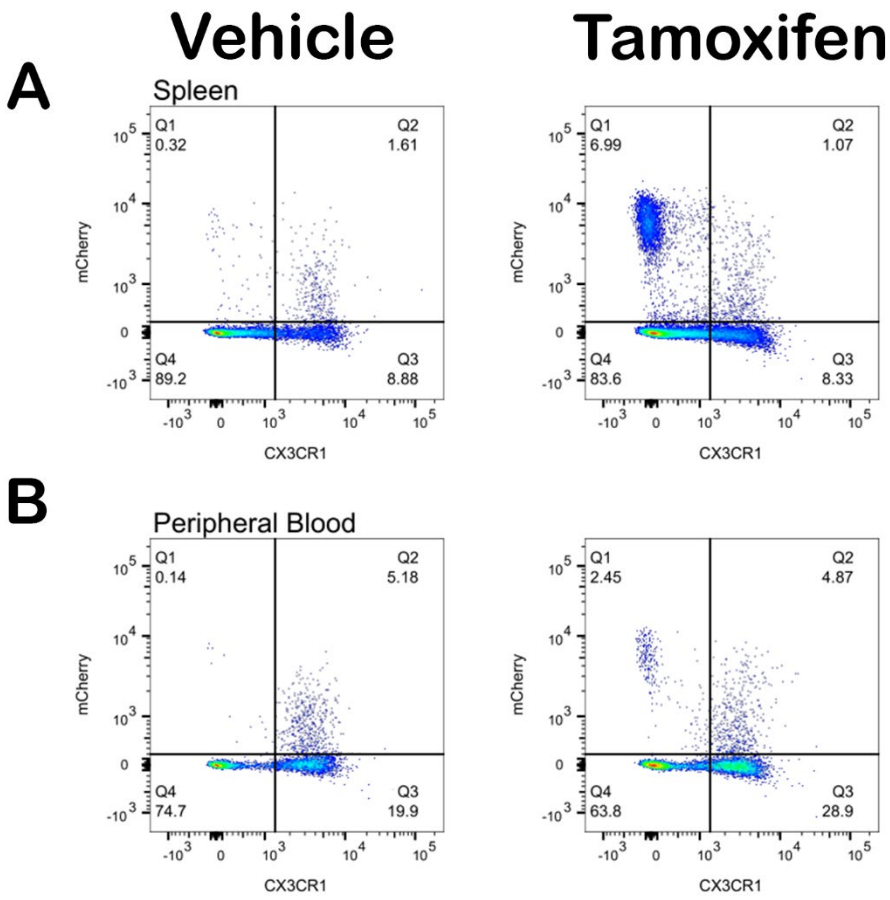

Figure S8: Flow cytometry gating comparisons for spleen and peripheral blood. Splenocytes (**A**) and peripheral blood (**B**) from either vehicle (left) or TAM (right) treated Cx3cr1-CreERT2$^{+/-}$; DIO-mCherry$^{+/-}$ were analyzed via flow cytometry. Representative gating charts are shown.

bioRxiv preprint doi: https://doi.org/10.1101/2025.07.17.665447; this version posted July 21, 2025. The copyright holder for this preprint (which was not certified by peer review) is the author/funder. This article is a US Government work. It is not subject to copyright under 17 USC 105 and is also made available for use under a CC0 license.

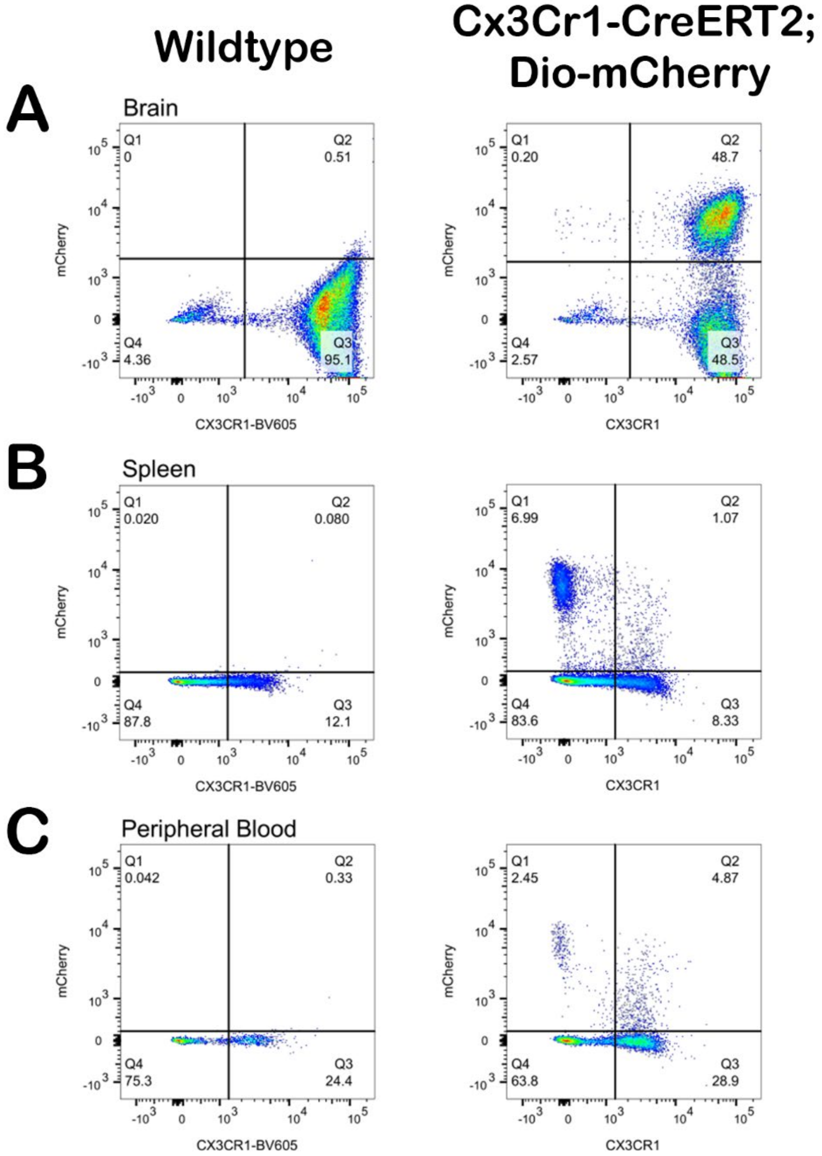

Figure S9: Flow cytometry gating determination of brain, spleen, and peripheral blood via stained WT negative controls.

TAM treated WT animals were used to gate thresholds for mCherry expression in brain (**A**), spleen (**B**), and peripheral blood (**C**) of TAM treated CreERT2$^{+/-}$; DIO-mCherry$^{+/-}$ rats.
